# Limitations and difficulties of echocardiographic short-axis assessment of paravalvular leakage after corevalve transcatheter aortic valve implantation

**DOI:** 10.1186/s12947-016-0080-5

**Published:** 2016-09-06

**Authors:** Marcel L. Geleijnse, Luigi F. M. Di Martino, Wim B. Vletter, Ben Ren, Tjebbe W. Galema, Nicolas M. Van Mieghem, Peter P. T. de Jaegere, Osama I. I. Soliman

**Affiliations:** 1 From the department of Cardiology, Thoraxcenter, Erasmus University Medical Center, Thoraxcenter, Ba304, ’s-Gravendijkwal 230, 3015 CE Rotterdam, The Netherlands; 2 From the department of Cardiology, Ospedali Riuniti, Università degli Studi di Foggia, Foggia, Italy; 3 From the Cardialysis Cardiovascular Core Laboratory, Rotterdam, The Netherlands

**Keywords:** Aortic valve, Regurgitation, Paravalvular, Echocardiography

## Abstract

To make assessment of paravalvular aortic leakage (PVL) after transcatheter aortic valve implantation (TAVI) more uniform the second Valve Academic Research Consortium (VARC) recently updated the echocardiographic criteria for mild, moderate and severe PVL. In the VARC recommendation the assessment of the circumferential extent of PVL in the short-axis view is considered critical. In this paper we will discuss our observational data on the limitations and difficulties of this particular view, that may potentially result in overestimation or underestimation of PVL severity.

## Background

**Fig. 1:**
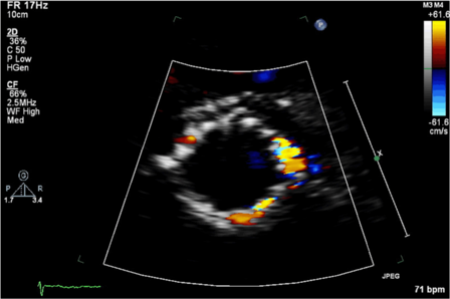
Multiple PVL jets seen at 2–3 o’clock, 4–6 o’clock, and 10 o’clock in the parasternal short-axis

**Fig. 2:**
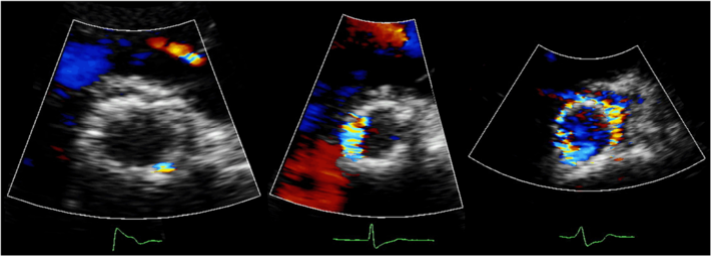
Examples of PVL severity according to VARC-2 criteria: mild <10 % (*left*), moderate 10–30 % (*middle*), and severe >30 % circumferential extent (*right*)

Transcatheter aortic valve implantation (TAVI) is a relatively new therapeutic option in patients with aortic stenosis (AS) who are at high-operative risk or inoperable [1, 2]. Despite its favourable hemodynamics, [2] paravalvular aortic regurgitation (AR) or leakage (PVL) after TAVI is common and is considered by many the Achilles’ heel of TAVI because there is growing evidence suggesting a significant association of PVL with short- and long-term mortality [3]. This may even become more troublesome as these therapies are offered to progressively younger patients. Unfortunately, the transthoracic echocardiographic assessment of PVL severity is extremely challenging. In contrast to AR in native valves, a zone of flow convergence and vena contracta, the single most important parameters in assessment of AR severity, [4, 5] do not exist or are difficult to image in patients with PVL. In contrast, the PVL is of variable size, starting somewhere at the level of the aortic annulus, running a poor to visualize course next to the stent frame with an exit of the flow into the left ventricle (LV) next to the inflow-end of the stent frame or into the LV “outflow tract”, in case of an eccentric jet passing through the stent frame before its end. In addition, even in native valves there has been no validation for adding multiple jets as may be frequently encountered in TAVI patients (Fig. 1). In the presence of a surgical aortic valve prosthesis it has been suggested that careful imaging of the neck of the jet in a short-axis (SAX) view, at the level of the prosthesis sewing ring, allows determination of the circumferential extent of PVL serving as a semi-quantitative measure of severity. According to this method a circumferential extent less than 10 % suggests mild PVL, 10 % to 20 % moderate PVL, and more than 20 % severe PVL (Fig. 2) [5, 6]. However, it should be noticed that this is only an approximate guide and its classification is strictly arbitrary, without any validation (the PVL volume of a given circumferential extent is unknown). Despite all the mentioned limitations and recognizing that all imaging windows should be used, the updated Valve Academic Research Consortium (VARC) adopted the SAX criterion as “critical” in assessing the number and severity of paravalvular jets in patients who underwent TAVI [7]. Surprisingly, in the most recent VARC-2 publication [8] the arbitrary cut-off value to define severe PVL was changed to more than 30 % (rather than the earlier published 20 %) [5–7] without any argument or reference given. This cut-off seems to be derived from a study published a year later, performed by some of the authors involved in the VARC2 publication [9]. Although we agree with the VARC-2 authors that – in absence of other simple and useful parameters – the SAX analysis is of vital importance in assessment of PVL it may result in significant underestimation or overestimation of PVL because of various reasons. Because we could in the literature not identify any detailed information on the specific problems of the SAX analysis we will in this expert opinion manuscript review the inherent limitations and difficulties of the SAX analysis in the estimation of PVL severity based on our observations.

### Data acquisition and analysis/Clinical characteristics

In total 554 transthoracic echocardiograms up to one year after implantation of a CoreValve Revalving System^©^ because of severe AS were analysed. These echocardiograms were acquired according to VARC recommendations in the period 2007–2013 [8]. More specifically, SAX images were scored for the presence of PVL jets according to a clock model [10]. Median age of the patients was 83 years, 46 % were males and body mass index was 26 ± 4. NYHA class 2, 3 and 4 before TAVI was present in 17 %, 63 % and 14 % of patients, respectively (the remaining patients were asymptomatic patients scheduled for cancer surgery). Consent was obtained obtained in all patients for anonymised prospective data collection for research purposes.

### False negative parasternal SAX imaging

**Fig. 3:**
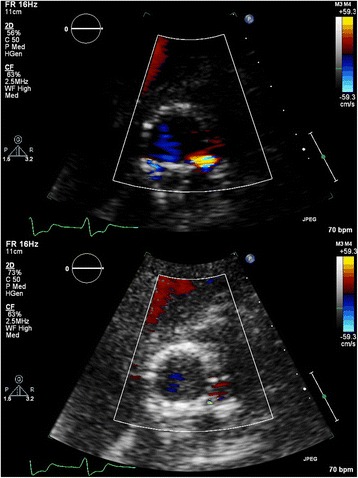
The destructive effect of 2D gain on colour Doppler. Top image gain 56 % and a clear jet seen at 5 o’clock, bottom image gain at 73 % with the same jet now barely visible

One of the major limitations in parasternal SAX analysis of the circumferential extent of PVL is the relatively high incidence of false negative studies, which may also imply underestimation of PVL in positive studies. In our series, 79 (14 %) of the SAX images were false negative, defined as absence of PVL in the SAX view but presence of PVL in the apical 3-chamber and/or 5-chamber views. Interestingly, in a recently published study it was shown that echocardiography (in contrast to angiography) did not correlate well with magnetic resonance in the assessment of PVL [11]. Echocardiographic analysis from a parasternal (long-axis) window underestimated AR with approximately 25 % of patients having a false negative study and 25 % having a positive but underestimated study. Also, in studies in which post-implantation angiographic and transthoracic parasternal echocardiographic data were compared the number of patients without PVL was clearly (up to three times) lower for echocardiography [12]. One of the inherent problems of parasternal imaging in post-TAVI patients is the imperfect imaging of the PVL jets due to acoustic shadowing by the stent or the crushed native material between the stent frame and the native aortic root or left ventricular outflow tract. In the normally positioned CoreValve prosthesis the new valve is positioned supra-annular, so the crushed native material will interfere with imaging of PVL jets distal to the new leaflet plane. In our series other clear reasons for false negative studies included poor quality in 5 % of the false negatives, SAX recorded at a too high level (valvular or supravalvular) in 8 % of the false negatives, and minor (trace) PVL (seen in apical views) in 43 % of the false negatives. Poor image quality due to patient characteristics like pulmonary disease (frequently present in the elderly referred for TAVI) or patient habitus and operator dependence (experience) are the Achilles’ heels of transthoracic echocardiography. Importantly, sonographers tend to increase the overall 2D gain in poor quality echocardiograms that further negatively influences colour Doppler imaging (Fig. 3). Interestingly, in patients with both a pre-discharge echocardiogram and follow-up study available, false negative studies occurred more often in the pre-discharge echocardiograms (28 % vs. 20 % at 6 months and 21 % at 12 months). One of the reasons for this difference may be the relative immobility of the patient shortly after TAVI precluding recordings in the left decubitus position.

### Localization of PVL

**Fig. 4:**
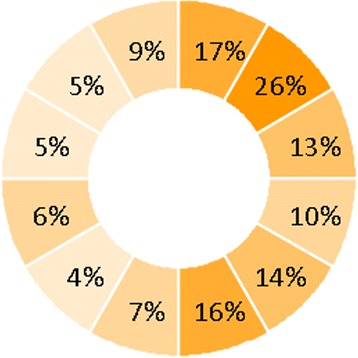
PVL localization in 223 consecutive pre-discharge SAX view﻿s according to a clock model

The localization of PVL jets in the SAX view has not been investigated in detail. According to Schultz et al. the localization of PVL is inversely related to the site of force of apposition of the stent frame to the adjacent tissue [13]. Thus, less PVL is seen at the outside curvature of the aorta because of higher apposition force, that may even be increased by a transfemoral route that may push the device toward the outside curvature of the aorta. In Fig. 4, the PVL localization in our series of consecutive pre-discharge echocardiograms can be seen. Most PVL jets were indeed seen at the site of lower force of apposition of the stent frame to the adjacent tissue at the inside curvature of the aorta, that is from 1 to 6 o’clock in the SAX image. Interestingly, PVL jets were most frequently seen at the commissures between the right and left coronary cusps (2 o’clock) and between the left and non-coronary cusps (5 to 6 o’clock). This may be caused by calcification nearby the commissures and subsequent creation of paravalvular commissural spaces next to the sites where the stent frame is sitting on the calcium ridges. Indeed, in one study it was shown that calcification in the area of the commissures predicted the site of PVL [14].

**Fig. 5:**
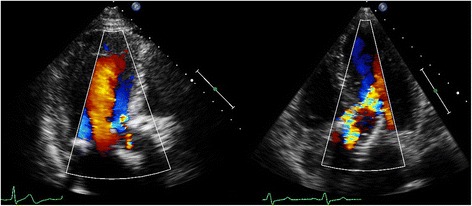
Two examples of eccentric PVL jets totally crossing over to the other side of the inflow part of the stent frame

Defining jet origin by the localization of the jet as seen just below the valve stent frame (as defined in the VARC paper) is prone to specific pitfalls. Firstly, the PVL jet origin at the level of the aortic annulus may be difficult to assess because we are actually imaging only the exit of the flow into the LV at the inflow-end of the stent frame. Secondly, many jets are eccentric, often passing through the stent before the inflow-end of the stent frame and some may even totally crossover to the other side of the inflow part of the stent frame (Fig. 5). Jets may at one extreme thus originate from the opposite site of the stent frame, although the incidence of totally crossover jets in our series was low (approximately 2 %). Also, *valvular* regurgitant jets may sometimes be eccentric and wall-hugging thus entering the LV from the inflow-end of the stent frame and falsely regarded as a localized PVL. These jets may in most cases be recognised because they follow a course along the inside of the stent frame. However, an isolated SAX view recorded just below the stent frame may make any eccentric jet, whether valvular or paravalvular, almost look similar.

### The level of SAX acquisition – “Flying jets”

**Fig. 6:**
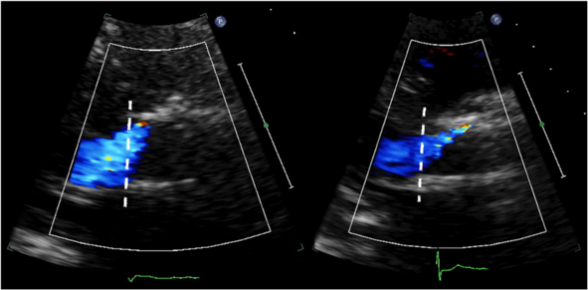
Two examples of eccentric PVL jets spreading out in all directions into the inflow area of the stent frame as seen in a parasternal long-axis view. Recording an image just below the stent frame (see dashed line) as recommended in the VARC publication will not show the “origin” or “neck” of the jet

**Fig. 7:**
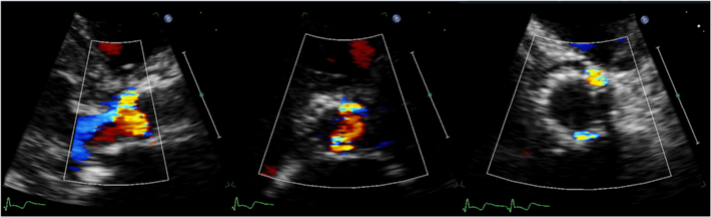
Parasternal long-axis showing two jets (*left*). Short-axis colour Doppler evaluation of PVL should start just below the valve stent (*middle*) *but should always be followed by* scanning more cranial to better detect the “origin” or “neck” of the PVL (*right*)

As mentioned earlier, according to the VARC recommendations colour Doppler evaluation of PVL should be performed just below the valve stent [8]. However, such an evaluation harbours apart from the earlier described problems in localizing the origin of PVL some other pitfalls. PVL jets are not simple jets running a straight course alongside the stent frame to the inflow part of the frame and the LV. In contrast, the jet will on its way towards the LV frequently encounter variously sized gaps or spaces, calcium ridges and crushed parts of the native aortic root and thus runs an unpredictable, variable (and difficult to visualise) course, and at certain levels it may even run in a more or less circumferential direction. In addition, as mentioned before, a substantial number of jets may pass through the stent frame before the inflow-end of the stent frame, and such an eccentric jet may spread out in all directions into the inflow area of the stent frame. Overestimation of PVL severity is therefore possible because obviously the true neck of the jet is in such cases not imaged at the recommended SAX level just below the stent frame (Fig. 6). Therefore, it should be recommended to start the SAX colour Doppler evaluation of PVL in a plane just below the valve stent *but always followed by* a more cranial scan to better detect the “origin” or “neck” of the PVL (Fig. 7).

**Fig. 8:**
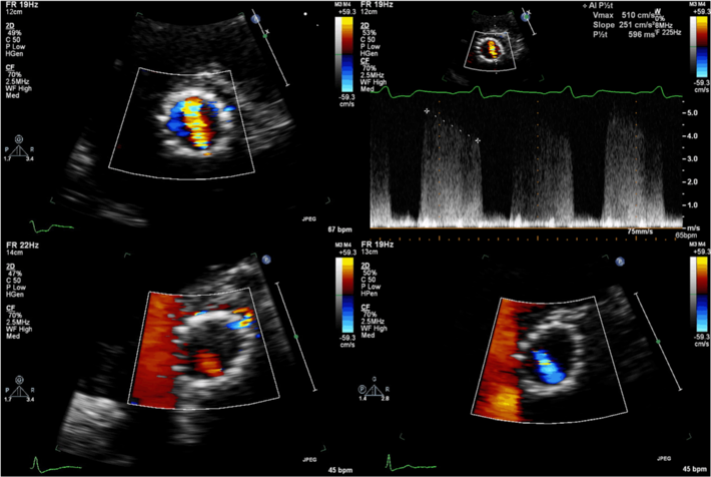
The origin of the PVL jet may be identified by Doppler imaging (*top images*) or the colour of the jet: red jet moving towards the transducer (*left, bottom image*) and blue jet moving away from the transducer (*right, bottom image*)

**Fig. 9:**
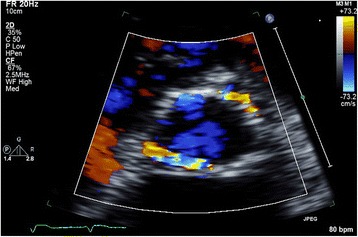
An extremely eccentric jet crossing the stent frame and mimicking a jet with an extensive circumferential extent. Note that it is actually a high velocity jet with a direction (“flying”) independent of the stent shape

Still it may not be possible to image the origin and neck of the jet in some patients, in particular in those with extremely eccentric jets that pass through the stent frame. In such cases the Doppler velocity may help to locate the origin of the jet (Fig. 8) and it may be better to estimate the circumferential extent of the jet by extrapolating its radius onto the circumference of the stent. In some cases the eccentric PVL jet is not mosaic coloured but red or blue coloured (Fig. 8); this will make the localization of the origin of the jet easy since a red jet will be directed towards the transducer (positive velocity on Doppler) and a blue jet will be directed away from the transducer, (negative velocity on Doppler). Problematic may, however, be that these low-velocity jets may contain less volume compared to a high-velocity jet giving an extra dimension to the difficulty in assessment of PVL severity. Another problem in eccentric jets that pass the stent frame is that occasionally they run a course into but directly next to the inflow part of the stent frame, giving the impression of a jet with an extensive circumferential area (Fig. 9). Again, the jet circumferential extent should be estimated by extrapolating the radial extent of the jet onto the circumference of the stent.

**Fig. 10:**
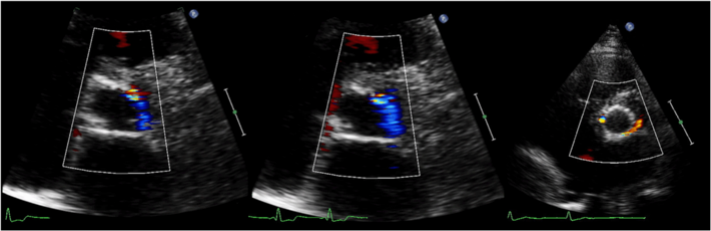
Overestimation of circumferential extent because the jet seems to follow a (partly) circumferential course. Left two images showing partly mosaic coloured jet and partly blue coloured. The jet presumably runs a circumferential course and the blue parts should be not regarded as circumferential extent. Left image shows a jet flying away from the stent, similarly the red parts should not be measured

Even more challenging may be the identification of parts of the jet in the SAX view that are actually not representative for the circumferential extent because the jet follows a (partly) circumferential course (Fig. 10). In such cases the circumferential extent of the PVL may be overestimated by giving in a SAX analysis the impression of an extensive jet. It is our practice to focus measurement on the mosaic part of the jet (Fig. 10). Typically red (jet directed towards the transducer, positive velocity on Doppler) or blue (jet directed away form the transducer, negative velocity on Doppler) parts are discarded. This should also be done in jets flying away from the stent (Fig. 10).

### Other problems in variability in jet numbers, localization and size

**Fig. 11:**
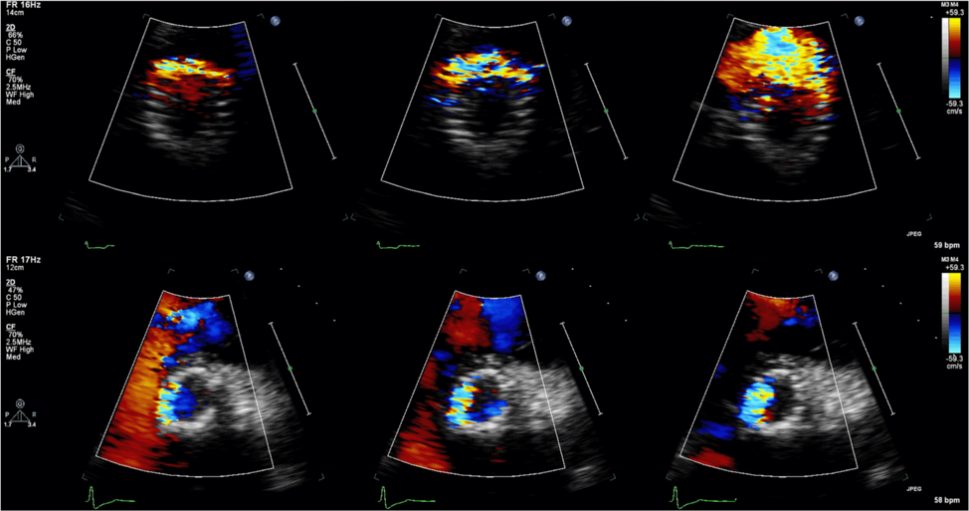
The circumferential PVL extent on SAX imaging may be quite variable within one beat dependent on the longitudinal motion of the heart

**Fig. 12:**
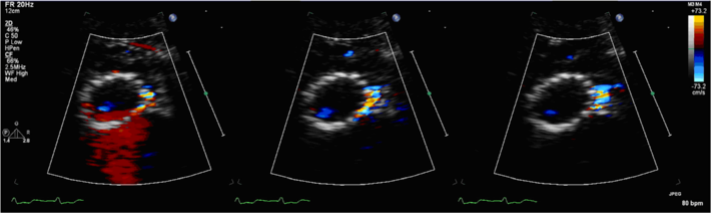
Variable circumferential PVL extent within one beat independent on the longitudinal motion of the heart

Another pitfall associated with the level of SAX imaging is the moment to measure the diastolic PVL because of the through-plane motion of the CoreValve prosthesis. In a normal contractile heart the longitudinal-orientated cardiac fibres of the heart cause the base of the heart to move towards the apex in systole whereas in diastole the reverse will occur: a motion of the base of the heart away from the apex [15]. Since the transducer is in a fixed position it means that in early diastole a relatively small jet may be captured but later in diastole maybe more colours are picked up because it represents a more distal level of the jet when it has spread in radial and circumferential directions (see Fig. 11). Also, the circumferential PVL extent on SAX imaging may be quite variable between cardiac beats and even within one beat independent on the longitudinal motion of the heart (Fig. 12). There is currently no recommendation on what time point in the cardiac cycle varying PVL size should be measured preferably. In our center we usually measure the third frame with visible PVL, unless this extent is clearly not representative as the average of the second to fifth frame with visible PVL (the first frame with visible PVL is always ignored because the jet is usually the smallest).

PVL jets at different locations may not only be seen in one beat but also in sequential phases of one beat or in different beats, as seen in a rocking prosthesis. This may overestimate the true severity of PVL, although in the case of a rocking prosthesis PVL severity is likely to be severe and not overestimated [16].

**Fig. 13:**
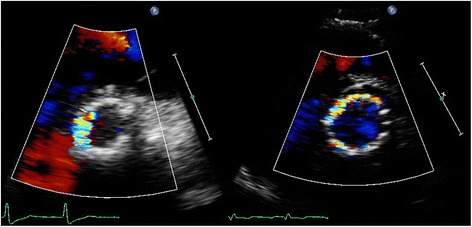
Severity of PVL assessed by circumferential versus radial extent. Left image shows a VARC score of 24 % versus right image 45 %. In contrast, the “area” of leakage is 1,23 cm^2^ versus 1,00 cm^2^, respectively

In surgical prosthetic aortic valves there may be a relatively fixed relation between the circumferential extent of valve dehiscence and the actual leaking area (defined by circumferential and radial extent). Because of the different pathophysiology (surgical dehiscence versus TAVI malapposition) this relation will be less clear in TAVI patients. This may result in the patient with a paradoxically high VARC score but limited radial extent that may actually have less PVL compared to a patient with a smaller circumferential extent but truly (see discussion in previous sections on level of image acquisition) larger radial extent (Fig. 13).

**Fig. 14:**
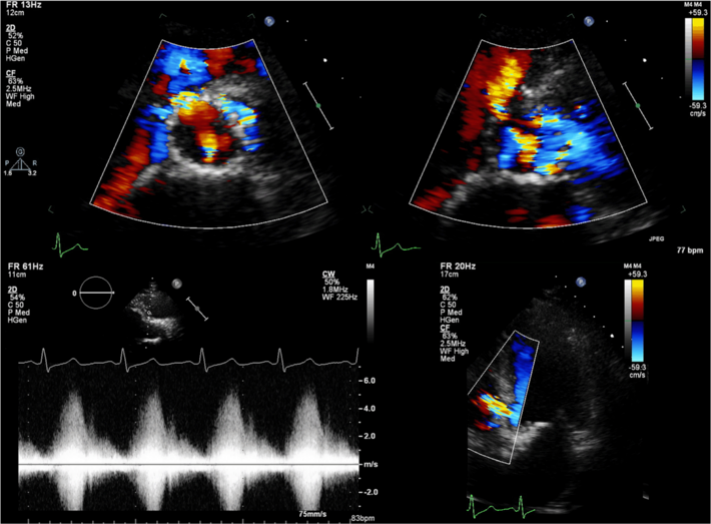
Ventricular septal rupture after CoreValve implantation mimicking PVL

A final reason for overestimation of PVL is the misinterpretation of a ventricular septal rupture after TAVI as PVL (Fig. 14) [17]. Careful colour and velocity Doppler imaging may easily differentiate between PVL and ventricular septal rupture.

### Limitations of our observations

In this paper the focus was on PVL after TAVI. Evaluating the presence and severity of AR should obviously include an assessment of both valvular and paravalvular components, with finally a combined measurement of ‘total’ AR reflecting the total volume load imposed on the LV. At current follow-up duration significant valvular AR is rare; in our institute it is present in a small minority of patients, and constitutes usually only a trace, with more than mild AR seen in the presented series in only a few patients with a not well deployed stent. However, with longer follow-up duration valvular AR will undoubtedly make the assessment of AR severity even more complex.

We reported only on our experience with the CoreValve Revalving System^©^, because our Edwards SAPIEN™ cohort is too small to provide a meaningful analysis. To what extent the described problems may occur in the Edwards SAPIEN™ prosthesis or other, newer percutaneous aortic valves should be evaluated - and compared to the CoreValve Revalving System^©^ - in future studies. Some of the discussed limitations (acoustic shadowing, imaging level) may be generalizable, whereas others may be more specific for the CoreValve Revalving System^©^. Of note, comparative papers on PVL severity between different percutaneous valves should be interpreted with caution. Comparing results from centres implanting different prostheses are significantly influenced by operator dependent PVL recordings and measurements with unknown inter-observer and inter-institutional variability. Comparing the performance of two different prostheses in one single centre should take in consideration patient/prosthesis selection bias. In our center the Edwards Sapien prosthesis is used mainly in patients with known conduction abnormalities (pre-existing right bundle branch block) or a sigmoid interventricular septum, and the latter may be predisposing to a higher incidence of PVL [12].

### Clinical implications and future perspectives

Studies that tried to predict the occurrence of PVL almost invariably divided PVL into less or more than mild. The methods to do so varied from abandoned echocardiographic methods to “according to VARC criteria”. Even the last method is acknowledged by the VARC authors to be “not well-validated” [8] and is prone to important limitations as described in this paper and by Pibarot et al. [10]. The high variability in the incidence of more than mild PVL in CoreValve specific studies (15 % to 34 %) [12, 18] and the variable correlation with angiography and magnetic resonance imaging [19] may be in part explained by the limitations in PVL assessment. Similarly, studies that related PVL to mortality should therefore also be interpreted with caution [3].

**Fig. 15:**
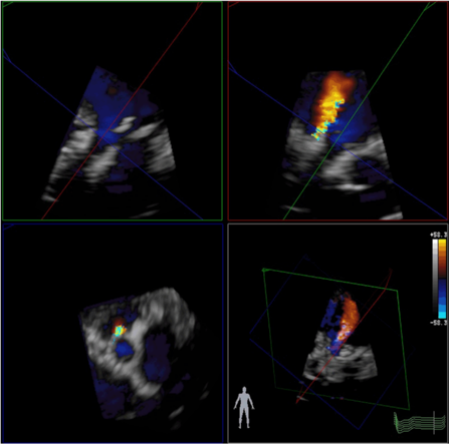
Assessment of the neck of PVL with three dimensional echocardiography
- Newly developed I-Rotate transducers do allow a full electronic rotation of 360° (adjustable by 5° steps) around the CoreValve prosthesis with excellent spatial and temporal resolution [21]. Similarly as studying the PVL extent of a mitral prosthesis with transesophageal echocardiography it may now be possible to study the PVL extent of a TAVI prosthesis by use of I-rotate colour Doppler in apical views, so avoiding interference with imaging of PVL jets by crushed native material or the stent (Fig. 16).

In our opinion there are several ways to improve echocardiographic assessment of PVL severity. Most importantly, sonographers and physicians should be aware of all the limitations of PVL assessment as described in this article and the final estimation of PVL severity should always integrate clinical data (symptoms and signs like the pulse-pressure and the Duroziez sign) with echocardiographic data (not only based on the circumferential extent but also on measures like the pressure half-time and backflow in the descending thoracic or abdominal aorta, although the value of these measurements in TAVI patients is not well established). In the future there are several ways to go:

- Use of a quantitative score model that incorporates apart from the SAX circumferential extent of PVL also the radial extent and measurements from standard apical views. These latter views may provide additional information, and in the TAVI population apical views are usually of better quality. Of note, in the apical views the exit of the PVL is seen without interference of the stent, because the latter is positioned deeper in the scan sector. Such a score was recently proposed by Pibarot et al. [10] - The role of 2D transesophageal echocardiography (usually well tolerated by the elderly) should be explored further because of its superior quality compared to transthoracic echocardiography.
- 3D colour flow imaging (Fig. 15) has by some been advocated as an accurate method to measure the vena contract of the PVL by imaging the SAX in a true perpendicular manner and imaging at the right level the “neck” of the PVL jet [20]. In our experience, the contribution of transthoracic 3D echocardiography to address the circumferential extent is rather low because of the lesser spatial (and temporal) resolution and the non-existence of a well-described neck in some patients. The role of 3D transesophageal echocardiography should be certainly explored further because of the better spatial resolution.

**Fig. 16:**
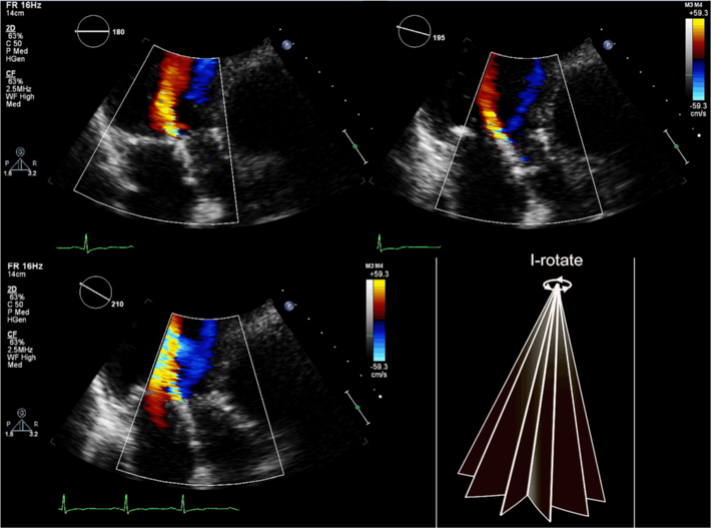
I-Rotate imaging in an apical window of PVL seen in a CoreValve prosthesis. Colour Doppler images are acquired at 15 degrees intervals

## Conclusion

The transthoracic echocardiographic SAX analysis of PVL is prone to important limitations and difficulties that may result in overestimation or underestimation of PVL severity. Future guidelines should incorporate these limitations and provide clear advices how to deal with them, in particular in case of eccentric jets and variability in jet size.

## Abbreviations

2D: Two dimensional
3D: Three dimensional
AR: Aortic regurgitation
AS: Aortic stenosis
LV: Left ventricle
PVL: Paravalvular regurgitation
SAX: Short-axis
TAVI: Transcatheter aortic valve implantation
VARC: Valve Academic Research Consortium
